# 1534. A Quality Improvement Initiative for HIV Prevention in Underserved Communities

**DOI:** 10.1093/ofid/ofad500.1369

**Published:** 2023-11-27

**Authors:** Meredith E Clement, Angie D Settle, Claudia T Martorell, Jeffrey D Carter, Melissa Rodriguez, Chelsie Anderson Chadha, Bonnie Douglas, Laura Simone, Leah Molloy

**Affiliations:** Louisiana State University Health Science Center–New Orleans, New Orleans, Louisiana; West Virginia Health Right, Inc., Charleston, West Virginia; The Research Institute, Springfield, Massachusetts; PRIME Education, LLC, Fort Lauderdale, Florida; PRIME Education, Fort Lauderdale, Florida; PRIME Education, LLC, Fort Lauderdale, Florida; PRIME Education, LLC, Fort Lauderdale, Florida; PRIME Education, LLC, Fort Lauderdale, Florida; PRIME Education, LLC, Fort Lauderdale, Florida

## Abstract

**Background:**

HIV prevention services like pre-exposure prophylaxis (PrEP) remain underutilized. This project aimed to support health care professionals (HCPs) in settings like federally qualified or community health centers and free and charitable clinics in expanding HIV prevention services for underserved populations, including immigrant communities.

**Methods:**

Baseline surveys were administered to interdisciplinary care teams in 6 clinics across the eastern US from January – March 2022. Survey data informed 4 live virtual education sessions featuring pre- and post-program surveys and team-based action planning. To promote exchange of clinical strategies, clinics with different levels of HIV prevention experience were paired together. Content was extended nationwide in an on-demand video.

**Results:**

Baseline survey respondents (N = 121) noted the greatest challenges in PrEP implementation were limited HCP knowledge/awareness (47%) and lack of training on PrEP workflows (33%). HCPs identified common barriers to patient PrEP uptake as lack of PrEP knowledge/awareness (58%), low perception of self-risk (47%), and low health literacy (38%). HCPs felt training was most needed in patient outreach/engagement (36%) and sexual history taking (32%), and 65% reported their team could most improve on normalizing sexual health conversations. 119 HCPs attended the education sessions (Table 1). HCP knowledge of laboratory monitoring for patients on PrEP rose from 40% to 57% (p = .037), as did the proportion of HCPs reporting high confidence in initiating/monitoring PrEP use (65% vs 93%, p < .001). Action plans included improving communication/care coordination (46%), increasing staff training/education (35%), and increasing patient outreach (35%) and education (35%). System champions from each clinic reporting 90-day outcomes (n = 16) agreed their clinics had improved in identifying patients for PrEP (94%) and evidence-based PrEP use (69%). As of March 2023, 1,531 HCPs had accessed the online video.

**Table 1.**
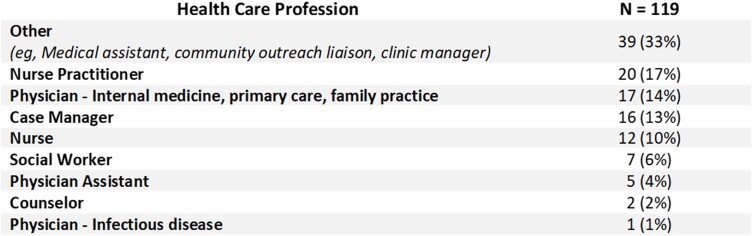
Education Session Attendees by Health Care Profession.

**Conclusion:**

HCPs gained confidence and knowledge in patient engagement and PrEP use and made action plans that helped improve PrEP implementation, emphasizing the potential impact of targeted education on HIV prevention services in underserved communities.

**Disclosures:**

**Meredith E. Clement, MD**, Gilead Sciences: Grant/Research Support|ViiV Healthcare: Advisor/Consultant **Claudia T. Martorell, MD MPH**, AbbVie: Honoraria|Gilead Sciences: Honoraria|GSK: Grant/Research Support|Theratechnologies: Grant/Research Support|ViiV: Honoraria

